# A Chinese Family With Adult-Onset Leigh-Like Syndrome Caused by the Heteroplasmic m.10191T>C Mutation in the Mitochondrial MTND3 Gene

**DOI:** 10.3389/fneur.2019.00347

**Published:** 2019-04-18

**Authors:** Tao-Ran Li, Qun Wang, Mao-Mao Liu, Rui-Juan Lv

**Affiliations:** ^1^Department of Neurology, Beijing Tiantan Hospital, Capital Medical University, China National Clinical Research Center for Neurological Diseases, Beijing, China; ^2^Department of Neurology, XuanWu Hospital of Capital Medical University, Beijing, China

**Keywords:** Leigh syndrome, Leigh-like syndrome, MELAS, MELAS-LS overlap syndrome, m.10191T>C mutation

## Abstract

Leigh syndrome (LS) is a mitochondrial disease of infancy and early childhood, that is rarely seen in adults. The high degree of genetic and clinical heterogeneity makes LS a very complex syndrome. The clinical manifestations include neurological symptoms and various non-neurological symptoms, with different mutations differing in presentations and therapies. The m.10191T>C mutation in the mitochondrial DNA gene encoding in the respiratory chain complex I (CI) subunit of MTND3 results in the substitution of a highly conserved amino acid (p.Ser45Pro) within the ND3 protein, leading to CI dysfunction and causing a broad clinical spectrum of disorders that includes LS. Patients with the m.10191T>C mutation are rare in general, even more so in adults. In the present study, we report a family of patients with very rare adult-onset Leigh-like syndrome with the m.10191T>C mutation. The 24-year-old proband presented with seizures 6 years ago and developed refractory status epilepticus on admission. She had acute encephalopathy accompanied by lactic acidosis, symmetrical putamen and scattered cortical lesions. The video electroencephalogram suggested focal-onset seizures. She harbored the heteroplasmic m.10191T>C mutation in her blood and fibroblasts. Her aunt was diagnosed with mitochondrial disease at the age of 42, and had the heteroplasmic m.10191T>C mutation in her fibroblasts. Her aunt's son (cousin) died of respiratory failure at the age of 8, and we suspected he was also a case of LS. Furthermore, we reviewed the previously reported patients with the m.10191T>C mutation and summarized their characteristics. Recognizing the characteristics of these patients will help us improve the clinical understanding of LS or Leigh-like syndrome.

## Introduction

Mitochondria are the sites of oxidative phosphorylation (OXPHOS) within eukaryotic cells, and mitochondrial diseases are a large group of genetic disorders that manifest with heterogeneous clinical features, characterized by defects in OXPHOS caused by mutations in genes that encode proteins involved in mitochondrial function (1, 2). The most common subgroups include mitochondrial encephalomyopathy with lactate acidosis and stroke-like episodes (MELAS), Leigh syndrome (LS), etc. Furthermore, a growing number of patients with mitochondrial disease exhibit overlap syndrome, such as MELAS/Leigh overlap syndrome (3–5), Alpers/Leigh overlap syndrome (6), etc.

LS is a devastating neurodegenerative disorder that was first described by Denis Archibald Leigh in 1951 (7). After a long period of research, the previously generally accepted concept was that LS is a neurodegenerative disease with variable symptoms due to mitochondrial dysfunction caused by a hereditary genetic defect accompanied by bilateral central nervous system lesions that can be associated with further abnormalities in diagnostic imaging (8). The term “Leigh-like syndrome” is used when these criteria are only partially met, or when patients present with atypical symptoms, laboratory findings, or radiologic features, but the overall clinical picture is highly suggestive of LS (9, 10).

LS occurs primarily in infancy and early childhood, affecting 1:32,000 to 40,000 newborns (11, 12). The onset of LS is typically within the first year of life and is often followed by rapid deterioration, with most patients dying within 5 years (13, 14), though a few patients survive for a longer period (15, 16). This disorder has been proven to be clinically and genetically heterogeneous. Clinical presentations include central or peripheral nervous system abnormalities and non-neurological abnormalities (10). To date, more than 75 disease-associated genes in mitochondrial DNA (mtDNA), as well as in nuclear DNA, have been identified in LS, along with various modes of inheritance (17). Although most patients have a nuclear DNA mutation, 25% of cases are caused by mtDNA mutations (18). Mutations primarily affect assembly factors or subunits of the mitochondrial respiratory chain, but mutation of genes involved in mtDNA replication, transcription, and translation have also been identified (19, 20). Furthermore, mutations have been found in proteins involved in other mitochondrial processes, or even non-mitochondrial processes, that can also lead to LS (19). In addition to mutant genes, the percentage of mutant mtDNA cells in an individual is a very important determinant in the pathogenesis, and the mtDNA mutation load must exceed a certain threshold to cause mitochondrial dysfunction (21, 22), which varies in different tissues and different mutations. This phenomenon leads to the observation that individuals with the same maternal inheritance may differ in clinical phenotypes. Even more perplexing, affected siblings who share the same mutation may present with different clinical features, suggesting a role for other genetic and/or environmental modifiers, and unrelated individuals with pathogenic mutations in different genes may share the same clinical phenotype (14, 23).

Rare cases of LS or Leigh-like syndrome can be observed in adolescents or adults, and these late-onset patients tend to present with atypical clinical features (24, 25). The patients with the T10191C mutation were reported in only a very few studies, and the clinical manifestations were varied (26). Here, we reported a family of patients with Leigh-like syndrome with the T10191C mutation; the proband was a 24-year-old female with a 6-year history of epilepsy, diagnosed as MELAS-LS overlap syndrome. Furthermore, we reviewed the characteristics of 28 patients with the T10191C mutation and summarized their characteristics, hoping to improve the clinical understanding of LS or Leigh-like syndrome.

## Case Report

**Figure 1:**
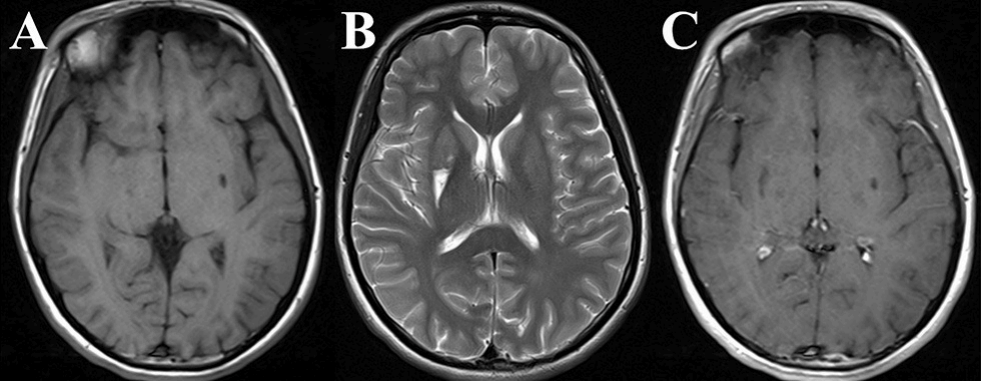
The proband's brain magnetic resonance imaging (MRI) obtained at 18 years old. Axial T1/T2-weighted images [Panels **(A)** and **(B)**, respectively] and contrast-enhancement T1-weighted image **(C)** of the brain were obtained when the proband appeared with the first seizures. An apparent large lesion was seen in the right putamen, and a relatively small lesion was seen in the left putamen.

The proband is a 24-year-old female and the only daughter born to unrelated and clinically unremarkable Chinese parents. There were no abnormal antenatal or postnatal issues of note, and her growth was normal. Her grades in school were below average, and her sports performance was ordinary. She obtained a junior college degree and was unmarried. She had previously been in good health, and the first concerns were raised when the patient was 18 years old. She developed paroxysmal unconsciousness and twitches affecting all four limbs after a cold, which subsided after about half a minute, with four similar attacks altogether. The brain magnetic resonance imaging (MRI) showed an abnormal signal in the T1/T2-weighted images of the bilateral putamen (Figure 1), and the magnetic resonance angiography was normal. She received 0.25 g of levetiracetam daily for a year, and seizures did not recur.

Six months ago, she developed paroxysmal shaking in her left limb and a prickling-like pain in her left forearm, which subsided after 5–10 s, with a frequency of more than ten times per day. The above seizure could be followed by a generalized tonic clonic seizure (GTCS). She received adequate doses of levetiracetam and lamotrigine, but there were still more than ten attacks per day. Twenty days ago, she experienced twitches in her face and tongue, with a frequency too high to be calculated. The frequency of attacks on the left side and right side is approximately 5 to 1. Initially, the twitches subsided within 5 s, but gradually extended to approximately 60 s each time. Four days ago, she had continual GTCSs and was diagnosed as status epilepticus (SE) in our hospital. She was given midazolam intravenously and levetiracetam, clonazepam and phenobarbital. Two days ago, she had not developed GTCS again, but still had frequent twitches in her face. In our epilepsy department, we adjusted the medication to 1.0 g of levetiracetam every 12 h, 2 mg of clonazepam every 12 h and 100 mg of phenobarbital daily. No twitches had reappeared as of 8 days later.

On admission, physical examination showed the patient was in a state of somnolence, and she could basically cooperate with the inspection. She exhibited dysarthria and left central facial and tongue paralysis. There was mild weakness and hypotonia in all four limbs. The tendon reflex was decreased bilaterally. Furthermore, the muscles of her four limbs exhibited mild atrophy, with the left side more obvious.

**Figure 2:**
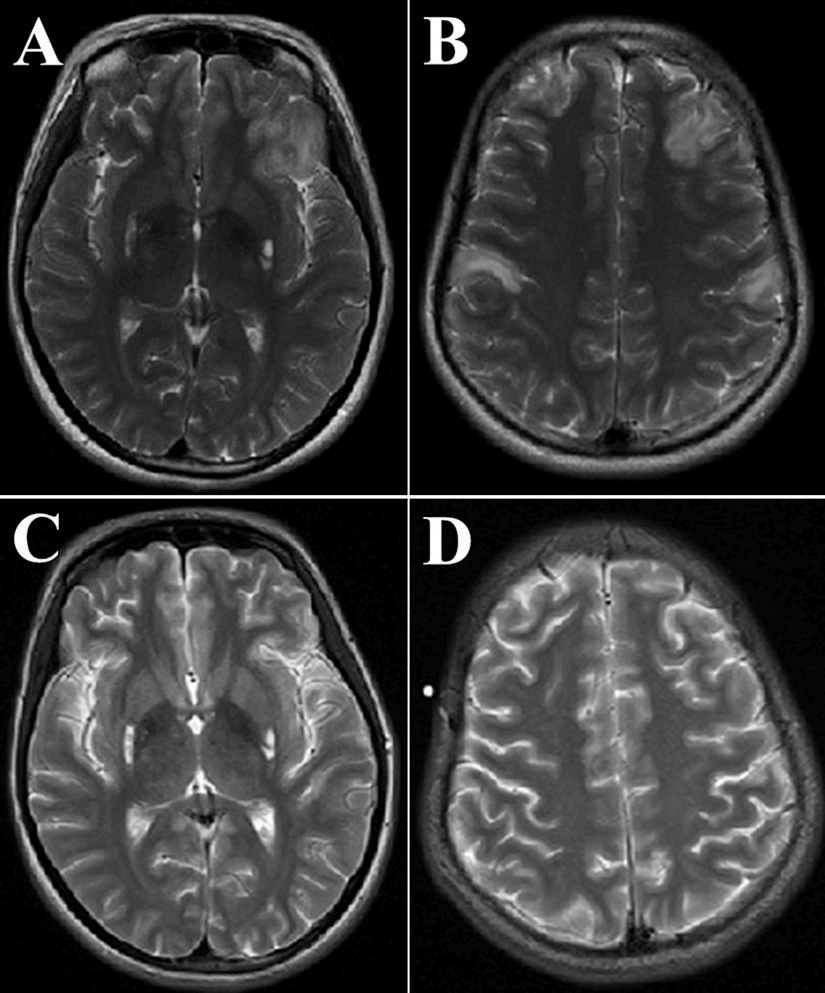
The proband's brain magnetic resonance imaging (MRI) obtained at 24 years old. Axial T2-weighted images of the brain MRI were obtained at the emergency department [Panels **(A)** and **(B)**, respectively], 24 days after treatment, the brain MRI was redone [Panels **(C)** and **(D)**, respectively]. Panels **(A,B)** showed frontal and parietal lobes cortical lesions and bilateral high-intensity areas in the putamen. Panels **(C,D)** showed that hyper signals in T2-weighted images of symmetrical putamen did not change significantly; however, the cortical lesions obviously improved.

A brain MRI showed hyper signals in the T2-weighted images of the symmetrical putamen, bilateral frontal and parietal cortices (Figures 2A,B). Twenty-four days after treatment, the cortical lesions had obviously improved (Figures 2C,D). Routine examinations of blood and cerebrospinal fluid (CSF) analysis were normal. The onconeural antibodies including anti-Hu, Yo, Ri, CV2/CRMP5, Amphiphysin, Ma2/Ta, recoverin, SOX1, titin, zic4, GAD65, and Tr (DNER), and the neuronal surface antibodies including anti-NMDA-R, CASPR2, AMPA1-R, AMPA2-R, LGI1, and GABA_B_-R in the serum and CSF were negative. Blood and urine metabolic screening were normal. There was one significant admission laboratory result: the blood lactate level was 4.5 mmol/L. The video electroencephalogram (EEG) showed there were significant diffuse slow waves in the right frontal lobe and temporal lobe, a small number of sharp waves in the right occipital and posterior temporal regions, and occasional sharp waves in the right temporal region during the interictal phase. During the ictal phase, we found the EEG rhythmic changes first appeared in the right frontal and anterior temporal regions, accompanying complex partial seizures. The brainstem auditory evoked potential, visual evoked potential, electromyography, and nerve conduction velocity were normal.

The Montreal Cognitive Assessment score was 25 on a scale ranging from 0 to 30. A biceps brachii muscle biopsy showed no ragged-red fibers (RRF). Under electron microscopy, there were damaged fibers in the striated muscle tissues and disorder arranged muscle nodes with increased glycogen and mitochondrial electron density.

**Figure 3:**
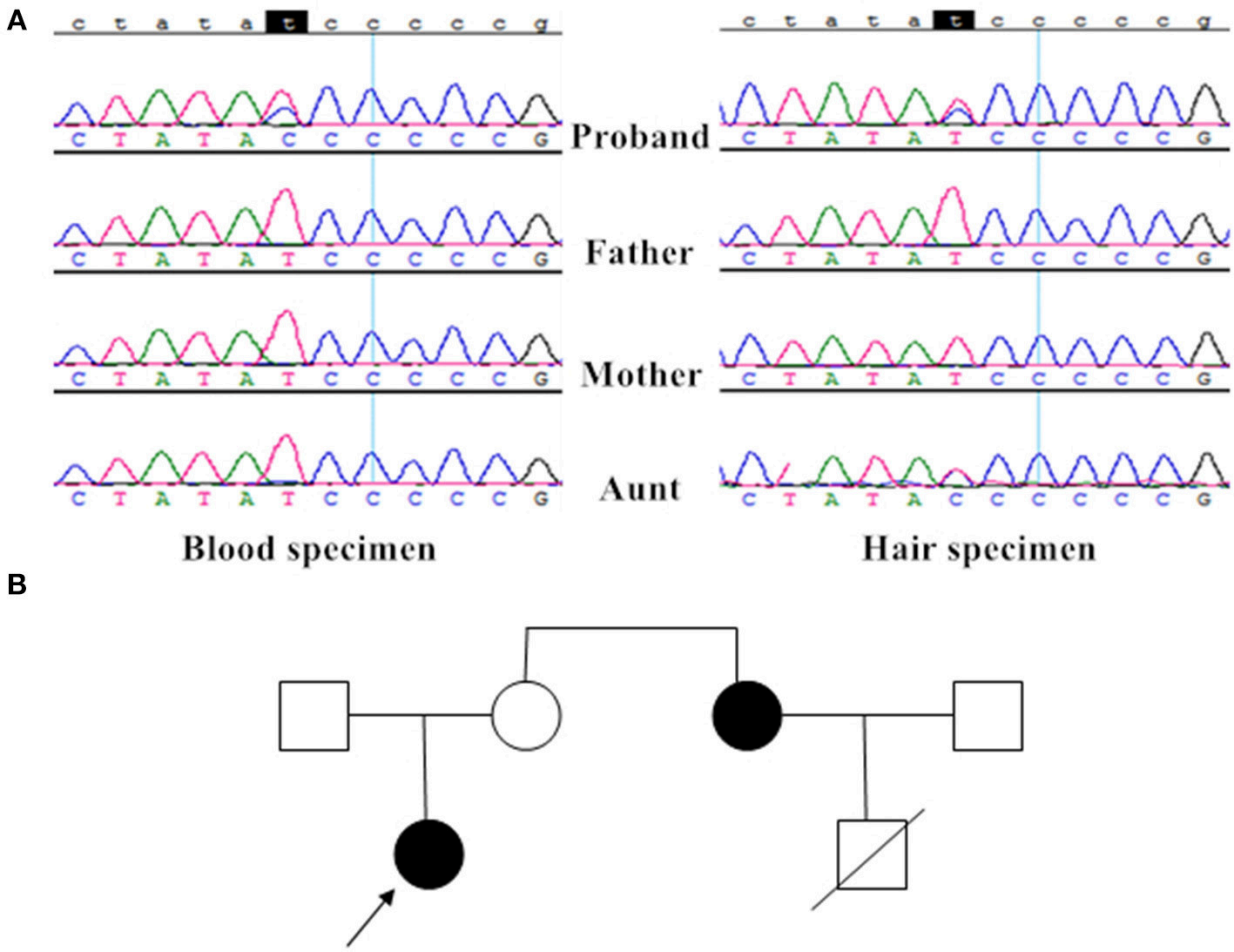
Mutation analysis and the family tree. **(A)** The proband had the heteroplasmic m.10191T>C mutation in blood and hair specimen, her aunt only had the heteroplasmic m.10191T>C mutation in her hair specimen; however, her parents had no detectable mutation. **(B)** The family tree showed two patients (the proband and her aunt) with the T10191C mutation.

Taking advantage of the PCR-Sanger sequencing technology in analyzing the mitochondrial genome, the patient was identified as having the m.10191T>T/C mutation in her blood and hair specimen; her aunt only had the m.10191T>C mutation in her hair specimen; however, her parents had no detectable mutation (Figure 3A).

The proband's aunt is 46 years old, with normal stature, hearing, and vision. She developed paroxysmal GTCSs at the age of 42. Her brain MRI showed multifocal lesions of the cortices at that time. She received carbamazepine 100 mg bid and gradually developed dysarthria. Three years later, she manifested twitches of the right side, followed by GTCS that occurred 4 times per day. Furthermore, she exhibited confused consciousness accompanied by occasional right extremity weakness. The brain MRI showed larger lesions than those that were present 3 years before, MR spectroscopy showed decreased N-acetylaspartate and a significant bimodal lactate peak (Supplementary Figure). Physical examination showed weakness of her right upper extremity (III/V). She was diagnosed with MELAS and given carbamazepine, levetiracetam and coenzyme Q10, as well as multivitamins. At present, she lives independently. The proband's aunt's son died of respiratory failure at the age of 8, but the details were not clear. The family tree is shown in Figure 3B.

All subjects gave written informed consents and written consent to permit publication of clinical details. The study was approved by the Medical Ethics Committee of Beijing Tiantan Hospital, Capital Medical University and was carried out in accordance with the Declaration of Helsinki.

## Discussion and Conclusion

The m.10191T>C mutation in the mtDNA of the complex I (CI) subunit of MTND3 results in the substitution of a highly conserved amino acid (p.Ser45Pro) within the ND3 protein, leading to CI dysfunction through impaired enzyme catalysis rather than impaired stability or assembly, causing a broad clinical spectrum of disorders (26). Patients with the m.10191T>C mutation are rare. In the present study, we report on a family of patients with the extremely rare adult-onset Leigh-like syndrome with the m.10191T>C mutation.

Including the two patients from our reported family, the m.10191T>C mutation has been reported in 28 patients to date (3, 5, 16, 26–44) (Table 1). There is a vast spectrum of clinical presentations and onset ages. According to the information presently available, the proportion of males is approximately 48%; the mean onset age ± standard deviation is 8.3y ± 10.7 y. In 2001, Taylor et al. first reported on a 42-year-old man with the m.10191T>C mutation who presented with a progressive history of epilepsy, stroke-like episodes (SLEs), bilateral optic atrophy, and cognitive decline (27); patient 2 was similar, with unremarkable clinical and MRI features that can only be identified as mitochondrial encephalomyopathy rather than any specific subtypes (33). The proband's aunt was diagnosed first with MELAS; however, we determined by genetic analysis that she has MELAS-LS overlap syndrome. The proband's aunt's son died of respiratory failure at the age of 8, so there was a substantial possibility that he was an undiagnosed LS patient. Consistent with the original researchers' viewpoints, 16/28 patients were diagnosed with LS, 9/28 were diagnosed with MELAS-LS, 2/28 were diagnosed with nonspecific encephalopathy, and there was insufficient information to evaluate patient 1. The characteristic imaging findings of LS were symmetrical hyper-intensities in the T2-weighted images. These lesions are commonly found in the basal ganglia or variable areas within the brain stem and several other regions (8). The cerebral cortex is not an area typically involved in LS. In this review, 13/28 patients presented with typical LS images, 9/28 patients presented with cortices in addition to typical lesions, 3/28 patients had mainly cortices involvement without symmetrical focus, patient 3 presented with a unilateral lesion, patient 18 did not undergo imaging examinations and the information on patient 1 was limited. In patients whose serum lactate levels were examined, 16/20 showed lacticemia.

**Table 1:** Patients with the m.10191T>C mutation.

**Patient**	**Onset age**	**Lacticemia**	**Adiitional features and autopsy**	**CT/MRI features**	**Family history**	**Muscle pathology**	**Gene mutation**	**Transmission**	**References**
1. U/U	U/U	U	U	U	U	U	T10191C	U	(38)
2. M/U	21 y/27 y	–	GTCS, focal motor EPC, myoclonic jerks, optic atrophy, sensory-motor deficit, cardiac arrest	Cerebral cortico-subcortical lesions	–	Morphologically normal; CI deficiency	95% T10191C mutation	U	(33)
3. M/U	24 y/alive at 42 y	Not Done	Migraine with aura, myoclonus, GTCS, ataxia, hypertonia, cognitive decline, optic atrophy, blind, downgaze, abnormal reflexes, SLEs	Midbrain, right superior colliculus, right lentiform nucleus	–	Increased range of fiber size, no RRF; no COX- negative fibers; CI deficiency	77% T10191C mutation, M; 14%, B	Maternal	(27)
4. F/MEL AS-LS	<16 y/alive at 18 y	–	Cognitive decline, migraine, short stature, DR, EPC, tingle, GTCS, nystagmus, anopsia, ataxia, myoclonic jerks	Perirolandic and calcarine cortices	–	Morphologically normal; high citrate synthase; CI deficiency	73% T10191C mutation, M; 19%, B	*De novo*	(37)
5. F/MEL AS-LS	11 y/alive at 19 y	+	Migraine with aura, myoclonus, GTCS, ataxia, ophthalmoplegia, dysarthria, SLEs, blurred vision, hearing loss, right-slanted tongue, right Babinski sign	Symmetrical basal ganglia and midbrain; bilateral parietal and occipital lobes	–	Morphologically normal; no RRF; no SSVs; no COX- negative fibers	T10191C	U	(39)
6. F/MEL AS-LS	10 y/alive at 26 y	+	divergent strabismus, nystagmus, hearing loss, dysarthria, weakness, involuntary movement, facial spasm, GTCS, abnormal reflexes, hypertonia (Japanese Writing)	Symmetrical putamen and scattered cortices	–	No RRF	T10191C	U	(3)
7. F/MEL AS-LS	16 y/U	+	Seizures, headache with flashing eyes, ataxia, SLEs, abnormal reflexes, hypertonia, right Babinski sign, hearing loss	Left frontal cortex, symmetrical red nucleus; right occipital lobe and left parietal lobe	–	Morphologically normal; no RRF	T10191C	U	(5)
8. F/MEL AS-LS	5 y/alive at 12 y	+	Intermittent tremor, ataxia, myoclonic seizures, dysarthria, hearing loss, SLEs, cognitive decline, mental alteration, weakness, focal motor seizures	Symmetrical basal ganglia, multifocal cortices	–	No RRF; no COX deficiency; mitochondrion normal; CI deficiency	T10191C, M	U	(36)
9. U/LS	U	+	DR, cognitive decline and others (Not given specifically)	Biateral brainstem and/or basal nuclei (Not given specifically)	+	CI deficiency	50% T10191C mutation, F and B; 90%, M	U (Maternal?)	(29)
10. M/LS	<1 y/U	+	DR, cognitive decline, ataxia, epilepsy, regression, SLEs, dystonia, progressive external ophthalmoplegia, optic atrophy, intrauterine growth failure	Leigh, stroke-like, thalamus lesions, white matter	U	Lipidosis; CI deficiency	T10191C	U	(41)
11. M/LS	1–16 y/U	–	Dystonia, DR	Leigh, cerebellar atrophy	U	Morphologically normal; no RRF; CI deficiency	T10191C, T10010C	U	(41)
12. F/MEL AS-LS	18 y/U	U	Cognitive decline, seizures, SLEs, ataxia, pyramidal syndrome, bulbar palsy	Bilateral basal ganglia and putamen; frontal, occipital, temporal, and parietal lobes; white matter	U	U	55% T10191C mutation, B	U	(44)
13. F/MEL AS-LS	16 y/U	U	Seizures, SLEs, ataxia, pyramidal syndrome, bulbar palsy	Bilateral midbrain, periaqueduct, super/infer colliculus, red nucleus, basal ganglia and putamen; frontal, occipital, and parietal lobes	U	U	52% T10191C mutation, B	U	(44)
14. F/LS	4–6 m/alive at 25 y	+	DR, cognitive decline, limited motility, upward gaze, dystonia, clonic seizures, GTCS	Bilateral basal ganglia, thalamus, midbrain, and periaqueductal gray matter; atrophy with ventricular dilation	–	fiber size variability; slight increase of perimysial connective tissue; no RRF; increased mitochondria; CI deficiency	70% T10191C mutation, F	Maternal	(16)
15. M/LS	6 y/alive at 16 y	+	Ptosis, weakness, motor regression, frequent falls, hypertonia, GTCS, thin, DR, cognitive decline, bilateral Babinski signs, abnormal reflexes	Symmetrical caudate nucleus and putamen	–	Mild lipid deposition; no RRF	T10191C mutation, B	U	(40)
16. F/LS	birth/U	+	Hypotonia, DR, myoclonic jerks, pyramidal syndrome, ophthalmoplegia	Symmetrical putamen	–	CI deficiency	90% T10191C mutation, M	*De novo*	(28)
17. M/LS	26 m/alive at 12 y	+	Cognitive decline, DR, drowsiness, vomit, myoclonic jerks, hypotonia, hypothermia, bradypnea, bradycardia; no speech and wheelchair bounded at 12y	Bilateral putamen, bulbar nuclei and globi pallidi, severe cortico- subcortical atrophy	–	CI deficiency	80% T10191C mutation, M	*De novo*	(28)
18. M/LS	birth/23 d	Not Done	DR, lethargy, hypotonia, abnormal reflexes, decreased muscle mass, micrognathia, equinovarus, bradycardic, respiratory acidosis, seizures. Autopsy	Not done	–	morphologically normal; no RRF; normal ATPase staining	100% T10191C mutation, F, K, S, L, and Br	*De novo*	(30)
19. F/LS	5 m/7 m	+	DR, dyspnea, hypotonia, dysphagia, fever, pneumorrhagia, coma	Bilateral cerebral hemisphere, basal ganglia, cerebellum, cerebellum, pons, and corpus callosum	–	Not Done	100% T10191C mutation, B	U	(43)
20. M/LS	5 w/U	–	DR, poor feeding, alertness, bradypnea, lethargy, hypotonia, clonus, abnormal reflexes, apnea, respiratory acidosis, lost all brainstem functions, seizures, spastic quadriplegia, decerebrate posture	Basal ganglia, midbrain, and pons	–	CI deficiency	100% T10191C mutation, M and B; 50%, F	Maternal	(32)
21. M/LS	6 y/alive at 16 y	+	Low birth weight, intrauterine embarrassment, apnea, DR, cognitive decline, epilepsy, hypertonia	Bilateral ganglia	–	Not Done; CI deficiency in blood	T10191C	U	(42)
22. M/LS	4 m/alive at 4 y	U	Hypotonia, DR, seizures	Bilateral basal ganglia subacute necrotizing lesions on MRI	–	Morphologically normal; no RRF; CI deficiency	68% T10191C mutation, M; 69%, B	*De novo*	(34)
23. F/LS	birth/alive at 22 m	+	Hypotonia, dysphagia, weakness, DR, ataxia, exercise intolerance, vomit, ophthalmoplegia, respiratory distress, partial seizures, GTCS	Symmetrical thalamus, cerebral peduncles	–	CI deficiency	97% T10191C mutation, M, F	Maternal	(31)
24. U/LS	13 m/alive at 11 y 9 m	U	Seizures, DR, spasticity, involuntary movement, gastrointestinal symptoms	Ventriculomegaly, atrophy, frontal gray matter, bilateral putamen, thalamus, brainstem	U	U	T10191C	U	(35)
25. M/LS	6 m/U	+	Macrocephaly, DR, ataxia, abnormal reflexes, spasticity, strabismus, seizures	Symmetrical putamen and head of caudate, right caudate body	–	CI deficiency	74% T10191C mutation, M; 65%, F	*De novo*	(26)
26. M/LS	birth/6 w	+	Poor feeding, lethargy, seizures, apnea, central hypotonia, peripheral hypertonia, weakness, abnormal reflexes, nonobstructive hypertrophic cardiomyopathy	Symmetrical brainstem extending to the lower medulla, basal ganglia, thalami, posterior limb of the internal capsule	–	CI deficiency	87% T10191C mutation, M; 64%, F; 87%, L	*De novo*	(26)
27. F/MEL AS-LS	18 y/alive at 24 y	+	Seizures, GTCS, and others	Symmetrical putamen, bilateral frontal and parietal cortices	+	No RRF; mitochondrial electron density increased	T10191C, F, B	Maternal	Current study
28. F/MEL AS-LS	42 y/alive at 48 y	U	GTCS, dysarthria, focal motor seizures, SLEs, weakness	Multifocal cortices, right cerebellar hemisphere, left thalamus	+	Not Done	T10191C, F	Maternal	Current study

U, unsure; COX, cytochrome c oxidase; EPC, epilepsia partialis continua; SLEs, stroke-like episodes; DR, developmental retardation; CI, Complex I; GTCS, generalized tonic-clonic seizure; RRF, ragged-red fibers; SSVs, strongly succinate dehydrogenase-stained vessels; M, muscle; B, blood; F, fibroblasts; K, kidney; S, spleen; L, liver; Br, brain.

Twenty-six patients (excluding patient 1 and 9) manifested abnormal motor findings, but the specific performance of each patient was different. Abnormal muscle tone was extremely common (21/26), 6/21 patients were hypertonic, 9/21 were hypotonic, 3/21 were dystonic, 2/21 were spastic, and 3/21 had pyramidal syndrome. Patient 16 presented with trunk hypotonia at birth and developed pyramidal syndrome later, patient 26 exhibited central hypotonia and peripheral hypertonia. We found infants tend to manifest as hypotonia, while the relatively older patients tend to manifest as hypertonia. Ten of the 26 patients had ataxia, 11/26 had dysarthria, dysphagia or other effects (bulbar palsy, no speech, or all brainstem functions lost), 11/26 had myoclonic jerks or other movement disorders (clonus, myoclonus, myoclonic, or clonic seizures, or facial spasm), 11/26 had weakness or other deficits (motor deficit, limited motility, wheelchair bound, or spastic quadriplegia), 2/26 had involuntary movement, and 1/26 had decreased muscle mass. Seizure was an extremely common symptom regardless of the age of presentation, and almost all patients (24/26, excluding patient 1 and 9) had seizures during the course of the disease; in some, seizures were even the initial presentation (patient 2, 7, 27, and 28) (5, 33). Developmental retardation was another common symptom (16/27, excluding patient 1); however, this may not be present in adult onset patients. A few patients had hypopsia (4/26, exclude patient 1 and 9) or were completely blind (27). Hearing loss can also be seen in MELAS-LS patients (4/26, excluding patient 1 and 9). SLEs appeared in 8/26 patients, mostly in MELAS-LS patients. Eleven of the 26 patients had ophthalmoplegia, strabismus or nystagmus. Four of the 26 patients had migraines or headaches, always accompanied by an aura. Ten of the 27 (excluding patient 1) patients had cognitive decline. Patients 8 and 20 manifested as mental alteration or alertness, respectively. Patients 17, 18, and 19 showed autonomic nervous disorders, mainly hypothermia, fever or bradycardia. In addition, a few patients appeared lethargic or even comatose in the late stages of the disease (26, 30, 32, 43). In addition to the above neurological manifestations, non-neurological abnormalities can also be present. In this case series, the most common symptoms were respiratory problems (7/26, excluding patient 1 and 9), which were very prominent in LS: 2 patients showed bradypnea (28, 32), 5 patients showed apnea or dyspnea or respiratory distress (26, 31, 32, 42, 43), 2 patients exhibited respiratory acidosis (30, 32), and the prominent initial symptom of patient 19 was pneumorrhagia. Other common non-neurological symptoms were gastrointestinal (5/26, excluding patient 1 and 9), consisting of vomiting and poor feeding (26, 28, 31, 32, 35). Patient 4 had short stature, patient 18 showed micrognathia and equinovarus, patient 25 was macrocephalic. One patient was diagnosed with non-obstructive hypertrophic cardiomyopathy (26).

In our reported family, the proband harbored the heteroplasmic T10191C mutation, and the prominent symptom was seizures until she was 18 years old. According to the presentation and EEG results, we confirmed that she had complex partial or focal onset seizures first, occasionally followed by secondary GTCSs. Researchers found 39.2% of patients had epileptic seizures throughout the disease course in 130 LS patients (13). Chevallier et al. enrolled 109 mitochondrial disease patients with a routine EEG examination, and concluded that 67/109 EEG studies were abnormal and 56/67 had epileptiform discharges. Furthermore, 8/16 LS patients had abnormal EEG findings, 7/8 patients had clinical or subclinical seizures with typical EEG abnormalities, and 1/8 patients only demonstrated diffuse slowing activity (45). In contrast, we found 92% of patients had seizures during the course of disease in this case series, so we believe that epileptic seizures could be typical manifestations of the T10191C mutation. Furthermore, no previous patients with the T10191C mutation had refractory SE such as our proband. Abnormal motor findings are frequent in LS patients, with an incidence as high as 99.2% (13), which was consistent with our findings. Hypotonia is the most common symptom among abnormal motor findings, and the proportion of 74.6% is far higher than our findings (42.8%) (13). Myoclonus and involuntary movement (chorea or athetosis) are evidently different from previous data, with incidence rates are 6.9 and 19.2%, respectively (13), compared to 42.3 and 7.7%, respectively, in our study. We speculate that one reason for this discrepancy is age difference; the median age of disease onset was 7 months, with 80.8% presenting symptoms by the age of 2 years, in reference (13), while the onset age is 8.3 ± 10.7 y in our study. Another reason could be that we recruited T10191C patients with high selectivity.

The proband's muscle biopsy revealed muscle injury and increased mitochondrial electron density. RRF, a hallmark of some mitochondrial diseases, is rare in LS (19) and is also not seen in our patient. In muscle biopsies of LS patients, a variety of changes in histologic and ultrastructural examination may be seen, such as lipid accumulation, COX-negative fibers, succinate dehydrogenase deficiency or abnormal mitochondrial configurations, or the muscle biopsy may be completely normal (13, 14, 19, 46).

Considering all patients with the T10191C mutation, particularly patients with high-ratio mutation (30–32, 43), we speculate that earlier onset and more severe phenotype are well correlated with both higher mutational load and lower residual activity of CI, which is in line with Malfatti et al.'s study (33). However, Nesbitt et al. thought the mutation loads measured in muscle and blood did not correlate with symptoms (26). Furthermore, patient 2, with a 95% heteroplasmic T10191C mutation, was healthy until the age of 21 years, so there must be other factors affecting the development of the disease (33). The proband and her aunt showed different mutation loads in their blood and hair samples, and, although her mother had no mutation detected in these samples, it does not mean she has no genetic mutation in other tissues or by repeated detection; we are convinced the hereditary mode of our family was maternal transmission, not *de novo* mutation. In addition, another 5 patients were suspected of maternal transmission (16, 27, 29, 31, 32), but we had the first reported family with definite diagnoses and clear genetic examination.

Our report also had some limitations. First, we did not find any mutations in the blood and hair samples of the proband's mother. Second, we did not determine the activities of the mitochondrial respiratory chain enzymes of our patients. Third, we can only obtain second-hand information, except for our two patients. Fourth, we combined patients of all ages due to the low incidence rate; however, the emphasis of physical examinations in infants or children are different from adults. Currently, there are no uniform criteria for the diagnosis of overlap syndrome, and researchers consider MELAS-LS overlap syndrome when LS patients develop characteristic appearance of MELAS. Therefore, the MELAS-LS overlap syndrome is also Leigh-like syndrome (9, 10).

Few diseases have such extensive genetic heterogeneity as LS, which has more than 75 monogenic causes (17). This genetic heterogeneity, together with highly variable phenotypes, low prevalence and early death, is challenging when performing large-scale clinical trials. The disease rarely occurs in adults, and adults with the T10191C mutation are reported in very few studies. We reported on a complete family with two adult-onset patients with the T10191C mutation who presented with epilepsy as the main clinical manifestation; furthermore, we reviewed previous patients with the T10191C mutation and summarized their characteristics. Recognition of the characteristics of these patients with the T10191C mutation will help us improve the clinical understanding of LS or Leigh-like syndrome.

## Ethics Statement

All subjects gave written informed consents and written consent to permit publication of clinical details. The study was approved by the Medical Ethics Committee of Beijing Tiantan Hospital, Capital Medical University and was carried out in accordance with the Declaration of Helsinki. We confirm that we have read the Journal's position on issues involved in ethical publication and affirm that this report is consistent with those guidelines.

## Author Contributions

R-JL, M-ML, and QW provided the family data. T-RL and R-JL acquired the literature data. T-RL drafted the manuscript. R-JL critically revised the manuscript for important intellectual content.

### Conflict of Interest Statement

The authors declare that the research was conducted in the absence of any commercial or financial relationships that could be construed as a potential conflict of interest.

## Supplementary Material

Supplementary Figure The aunt's brain magnetic resonance imaging (MRI) obtained at 43 years old. Axial images of the brain were obtained during her second hospitalization [Panels **(A–E)**, respectively]. **(A–E)** The T2-weighted fluid-attenuated inversion recovery images showed high-intensity areas in the left frontal lobe, bilateral parietal lobes, bilateral temporal lobes, left occipital lobe, left corpus callosum, and the brainstem was obviously compressed. **(F)** The MR spectroscopy analysis of the red area showed decreased N-acetylaspartate and a significant bimodal lactate peak.
