# Addendum: A Chinese Family With Adult-Onset Leigh-Like Syndrome Caused by the Heteroplasmic m.10191T>C Mutation in the Mitochondrial MTND3 Gene

**DOI:** 10.3389/fneur.2019.00936

**Published:** 2019-09-18

**Authors:** Tao-Ran Li, Qun Wang, Mao-Mao Liu, Rui-Juan Lv

**Affiliations:** ^1^Department of Neurology, Beijing Tiantan Hospital, Capital Medical University, China National Clinical Research Center for Neurological Diseases, Beijing, China; ^2^Department of Neurology, XuanWu Hospital of Capital Medical University, Beijing, China

**Keywords:** Leigh syndrome, Leigh-like syndrome, MELAS, MELAS-LS overlap syndrome, m.10191T>C mutation

In the original article, there was an error. There were two spelling mistakes in the article.

A correction has been made to **Discussion and Conclusion**, last sentence of Paragraph 8:

“Recognition of the characteristics of these patients with the T10191C mutation will help us improve the clinical understanding of LS or Leigh-like syndrome.”

The authors apologize for this error and state that this does not change the scientific conclusions of the article in any way. The original article has been updated.

## Addendum

In the original article, we had not obtained the heteroplasmy levels of the proband and her aunt from the gene sequencing company at the time of manuscript publication.

Through follow-up research, we make the following addendum:

By detecting the proband's and her aunt's blood sample, we found the heteroplasmy level of the proband is 33.2% (forward percent) or 38.2% (reverse percent) respectively, the heteroplasmy level of her aunt is 4.4% (forward percent) or 4.8% (reverse percent), respectively.

