# Supplementary figures and images for: A Chinese Family With Adult-Onset Leigh-Like Syndrome Caused by the Heteroplasmic m.10191T>C Mutation in the Mitochondrial MTND3 Gene
Source: Front Neurol. 2019 Apr 18;10:347. doi: 10.3389/fneur.2019.00347 (PMC6499163; doi:10.3389/fneur.2019.00347)

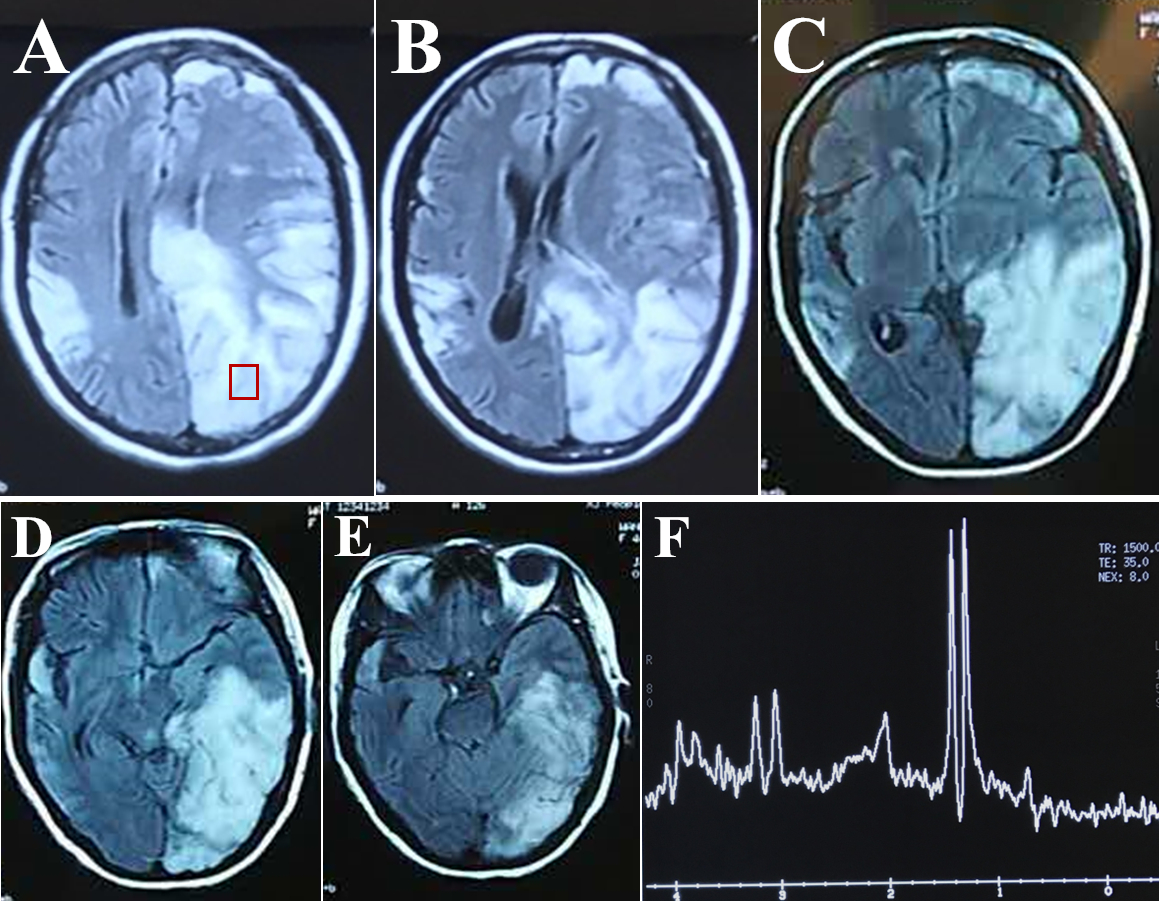

Supplement: Supplementary Figure — The aunt's brain magnetic resonance imaging (MRI) obtained at 43 years old. Axial images of the brain were obtained during her second hospitalization [Panels (A–E), respectively]. (A–E) The T2-weighted fluid-attenuated inversion recovery images showed high-intensity areas in the left frontal lobe, bilateral parietal lobes, bilateral temporal lobes, left occipital lobe, left corpus callosum, and the brainstem was obviously compressed. (F) The MR spectroscopy analysis of the red area showed decreased N-acetylaspartate and a significant bimodal lactate peak. [file Image_1.JPEG]
